# Computers and chess masters: The role of AI in transforming elite human performance

**DOI:** 10.1111/bjop.12750

**Published:** 2024-12-05

**Authors:** Merim Bilalić, Mario Graf, Nemanja Vaci

**Affiliations:** ^1^ Northumbria University at Newcastle Newcastle UK; ^2^ Alps Adria University Klagenfurt Austria; ^3^ University of Sheffield Sheffield UK

**Keywords:** AI, Bayesian analysis, generalized additive models (GAM), longitudinal study, multiple change point (MCP)

## Abstract

Advances in Artificial Intelligence (AI) have made significant strides in recent years, often supplementing rather than replacing human performance. The extent of their assistance at the highest levels of human performance remains unclear. We analyse over 11.6 million decisions of elite chess players, a domain commonly used as a testbed for AI and psychology due to its complexity and objective assessment. We investigated the impact of two AI chess revolutions: the first in the late 1990s with the rise of powerful PCs and internet access and the second in the late 2010s with deep learning‐powered chess engines. The rate of human improvement mirrored AI advancements, but contrary to expectations, the quality of decisions mostly improved steadily over four decades, irrespective of age, with no distinct periods of rapid improvement. Only the youngest top players saw marked gains in the late 1990s, likely due to better access to knowledge and computers. Surprisingly, the recent wave of neural network‐powered engines has not significantly impacted the best players – at least, not yet. Our research highlights AI's potential to enhance human capability in complex tasks, given the right conditions, even among the most elite performers.

Yes, I have been influenced by my hero AlphaZero recently. Essentially, I have become a very different player in terms of style than I was before, and it's been a great ride. Magnus Carlsen, June 2019

## BACKGROUND

Technology has always played a pivotal role in reshaping the way people live, from the inception of electricity, which brought light to homes and powered machinery, to the invention of personal computers, which revolutionized how we process information and communicate. Arguably, the most important recent technical development is the advent of Artificial Intelligence (AI), a set of procedures and rules that produce intelligent output not unlike what humans do, in the middle of the last century. A recent explosion in machine learning (Sarker, 2021) and large language models (Thirunavukarasu et al., 2023) has led to a paradigm shift in numerous sectors, making the influence of AI on human performance omnipresent. The benefits of AI are, however, debated as it is difficult to gauge the full extent of technology's impact on human behaviour. Here we demonstrate that AI has profoundly changed the behaviour of the best chess players, who markedly improved their performance at two points in history: (1) in the late 1990s with the availability of personal computers (PCs) and the advent of the internet and (2) in the late 2010s with the influx of chess engines based on deep neural networks. Differently aged and skilled players were, however, differently impacted by the two AI milestones. The findings in our study are a direct product of the AI‐like procedures which have catalysed improvements in real‐world domains. Our study not only reinforces our understanding of AI's impact on an intellectual realm but also provides an example of its prospective applications in the field of psychology.

### AI advances and human performance

Technical advances are often followed by fear (Pycha et al., 1986). This is in particular the case with AI where even the name of the new technology states that it is about machine intelligence which matches human apparently intelligent output. We define AI here broadly as development of computer systems capable of doing tasks that are normally thought to require human intelligence, be it making decisions, solving problems, or recognizing speech (Nilsson, 1982). Recent technological advancements led to machines being able to generate text, understand images, and in general learn from available data (Wang et al., 2023). This inevitably led to a paradigm shift in numerous sectors and inevitable fear of whether the AI will replace humans as decision‐makers (Mollick, 2024).

Here we are, however, more interested in exploring a less pessimistic, and we would argue more realistic, aspects of humans learning with and from AI. Successful learning normally requires immediate feedback (Lipnevich & Panadero, 2021), a process that becomes more complex in fields where performance is interdependent on interactions with other actors (Levine et al., 2017). In such scenarios, conventional methods like books, lectures, or even specialized coaching may not suffice (Gaessler & Piezunka, 2023). The alternative, involving interaction with a human training agent, though more efficient, often faces constraints due to availability (Gaessler & Piezunka, 2023). It is in this context that the latest advancements in AI prove invaluable. AI simulations, capable of dynamic adaptation to the learner's needs, offer a realistic emulation of training interactions. From medicine and healthcare to finance and business, AI's application extends to various domains for tailor‐made, interactive, and effective learning experiences (for a review, see Lai et al., 2023).

For example, Noy and Zhang (2023) discovered that integrating ChatGPT significantly enhances productivity in college‐educated professions by reducing the time needed for tasks by 40% and improving output quality by 18%. Similarly, Dell'Acqua et al. (2023) demonstrated that AI integration in a professional's workflow, specifically for tasks within AI's capabilities, leads to notable performance gains. In an analysis of 18 realistic business tasks, AI utilization resulted in over 25% faster task completion, more than 40% improvement in human‐rated performance, and a marked increase in task completion rates. These results highlight the transformative potential of AI in professional settings. Both studies, however, emphasized that AI's effectiveness depends on its alignment with task requirements and the skill level of the human participants. Lower‐skilled individuals benefited most from AI assistance, and performance sometimes even decreased when tasks exceeded AI's current capabilities (Dell'Acqua et al., 2023).

Statistically, highly skilled individuals are less likely to show significant improvement due to the ceiling effect. Their performance is already at a high level, leaving little room for further enhancement. This restriction in the range of data often leads to suppressed effects (Pearson, 1902; Vaci et al., 2014). Nonetheless, a key insight from these studies is that AI needs to be sophisticated enough for high‐performing individuals to derive additional value. We now shift our focus to the realm of chess and elite chess players to explore whether AI can indeed offer value in such high‐skilled environments if the level of AI matches or exceeds that of best human performers.

### Chess as drosophila in both psychology and AI

Beginning with the investigations of intelligence pioneer Alfred Binet into mental imagery (Binet, 1894), and extending through Adriaan de Groot's seminal work on cognitive processes (Degroot, 1978), to Nobel laureate Herbert Simon's studies on decision‐making (Chase & Simon, 1973a; Gobet & Simon, 1996), chess has often been used as a domain of research by psychologists. It presents a deceptively simple set of rules within a complex environment, making it a particularly suitable ground for experimental inquiry–so much so that it has been dubbed the ‘Drosophila of cognitive science’ (Chase & Simon, 1973b). One of its most attractive attributes is the existence of an objective metric of skill, the Elo rating system (Elo, 1978; Vaci & Bilalić, 2017), a feature unique to chess, where players' rankings are determined exclusively by their performance against other competitors. This characteristic has enabled researchers to utilize chess as a means to explore not only quintessential cognitive science themes such as cognitive processes (Bilalić et al., 2008b; Bilalić, McLeod, & Gobet, 2009; Campitelli et al., 2005, 2007; Chassy et al., 2023; Gobet, 2000; Gobet & Simon, 1996; Moxley et al., 2012) but also broader psychological phenomena including gender disparities (Arnold et al., 2023; Bilalić, Smallbone, et al., 2009; Vishkin, 2022), stereotype threat (Backus et al., 2023; Smerdon et al., 2020; Stafford, 2018), individual differences (De Bruin et al., 2005; Waters et al., 2002) and the enduring nature versus nurture debate (De Bruin et al., 2014; De Bruin et al., 2008; Vaci et al., 2019).

In recent years, scientists have begun to exploit a more granular aspect of chess–individual moves within each game. These discrete moves represent a sequence of individual decisions, the quality of which can be compared with an objective, context‐independent benchmark: the superior decisions made by chess engines in analogous situations. This comparison affords a more precise and objective means of assessing the performance of players across different historical epochs. Utilizing this microanalytic approach, researchers have explored a variety of inquiries, such as the cognitive impact of wearing masks (Smerdon, 2022), the decision‐making quality fluctuation over decades and the influence of emerging technologies on it (Gaessler & Piezunka, 2023), and whether gender‐related disparities in error rates during play–potentially attributable to stereotype threats–exist between female and male opponents (Backus et al., 2023). Our current study contributes to this body of work by employing the micro‐level analysis of chess moves to examine the effects of AI advancements on human performance.

The field of AI, since its inception, has frequently employed chess as a prototypical research domain, with some referring to it as the ‘Drosophila of AI’ (McCarthy, 1990). Its well‐defined, simple rules and confined space allow for a clear mathematical formalization, as illustrated in the seminal works of Newell et al. (1958) and Shannon and Shannon (1950). Yet, beneath this facade of simplicity, chess is a game of immense complexity. It is postulated that the game's possibilities exceed even the number of atoms in the known universe, with the legal positions in chess estimated at 10^40^ and the number of different possible games at 10^120^, while the number of atoms in the universe is estimated at 10^75^. To draw a more vivid comparison, a player envisioning eight moves ahead is faced with a number of possible continuation equivalent to the stars in the galaxy (Rasskin‐Gutman, 2009). Chess is thus acknowledged as an intellectual domain par excellence, demanding the full spectrum of higher‐order cognitive functions such as reasoning, thinking, and problem‐solving–core attributes that define human intelligence. Mastery of chess, and competent play, has been seen as a demonstration of human‐like performance within a domain of great complexity. This challenge has positioned chess as the quintessential arena for the pursuit of AI's ultimate ambition (Ensmenger, 2012).

The fascination with chess as a means to conceptualize artificial intelligence is epitomized by the works of Alan Turing, widely regarded as the forerunner of theoretical computer science and artificial intelligence, and Claude Shannon, esteemed as the father of information theory. Turing envisioned a chess‐playing machine as emblematic of a thinking machine (1946), and his subsequent conjectures regarding the potential for ‘machine intelligence’ predominantly revolved around the concept of a chess‐playing computer (Turing, 1953, 2009). He was the first to theoretically articulate the concept of a computer capable of playing chess (Turing, 1946). Nonetheless, it was Shannon who undertook the initial comprehensive analysis of computer chess. He viewed the game as the quintessential test bed for AI, attributed to its simultaneous simplicity and complexity. Shannon's theoretical work marked a crucial turning point. Though Shannon acknowledged the objective of AI to emulate human cognition in intricate domains like chess, the hypothetical chess computer he proposed utilized a decidedly non‐human, brute‐force strategy. Unlike humans, who would concentrate on a limited set of promising possibilities (Bilalić et al., 2008a; Degroot, 1978), Shannon's theoretical machine was designed to perform an exhaustive, tree‐like search (Shannon & Shannon, 1950). This model was refined by computer and cognitive science vanguards Newell, Shaw, and Simon, who developed the first functional computer (1958) capable of playing chess by enhancing Shannon's algorithms (for a review, see McCarthy, 1990), thus inspiring Simon's bold assertion that a digital computer would defeat the world chess champion within a decade (Simon & Newell, 1958). While this straightforward approach was enticing and mathematically executable, it yielded suboptimal outcomes, a consequence of the limitations in hardware and the inherent complexity of the domain. As a result, Simon's late‐1950s predictions that computers would rival the finest human chess players within a decade proved to be excessively optimistic.

The brute‐force approach held significant appeal for early AI developers. With progressively refined evaluation functions and advanced search algorithms, coupled with the burgeoning availability of computational resources, Simon's prediction of machines surpassing human chess capability ultimately materialized in the late 1990s. In 1997, an IBM computer named Deep Blue, which incorporated algorithms akin to those proposed by Shannon and enhanced by Newell and Simon, triumphed over the reigning world chess champion, Garry Kasparov, in a historic six‐game match (Hsu, 2002). The victory was largely attributed to the hardware's capability to calculate 200 million moves per second, showcasing a markedly non‐human modus operandi. Thus, a pursuit quintessentially associated with human intellect was surmounted using a distinctly non‐human strategy, characterized by profound search depth and rapid pruning techniques (Hsu, 2002; Kasparov, 2017).

In the domain of computer science, a paradigm shift occurred during the mid to late 2010s with the advent of deep neural networks based on deep reinforcement learning, marking a departure from traditional brute‐force methods. This new breed of algorithms diverges significantly from its predecessors, such as the minimax and alpha‐beta pruning techniques. While the latter rely on extensive search trees to anticipate opponent moves, deep neural networks operate on a different principle. They are designed to parse vast datasets, learning from a wealth of prior knowledge or completely from scratch, identifying patterns and strategies that even the most comprehensive brute‐force searches may not uncover.

The prowess of deep reinforcement learning was epitomized by AlphaGo, an AI engine that utilized this sophisticated learning approach to defeat the world's premier Go player (Silver et al., 2016). However, the innovation did not culminate with AlphaGo. The subsequent iteration, AlphaZero, embarked on learning the game of Go from the ground up, without any prior human knowledge, achieving even more remarkable results than its predecessor (Silver et al., 2018). Unlike specialized systems such as Deep Blue, which was tailored exclusively for chess, the learning mechanisms underpinning AlphaZero were generalized, enabling its application across various domains, not only chess and Go but also video games (Vinyals et al., 2019). This approach's versatility was further showcased by AlphaFold, an AI developed by the same principles, which has made significant strides in the field of biology (Jumper et al., 2021).

### Human (chess) performance through history

In domains where knowledge grows and spreads, new generations may naturally outpace their forebears. Chess computers and engines epitomize tools for expedited knowledge sharing. They serve not merely as ever‐available sparring partners but also as repositories of chess wisdom, particularly beneficial when human opponents are less skilled than the silicon training partners. It is a reasonable supposition that chess computers have enhanced the performance of the average player. A recent study (Gaessler & Piezunka, 2023) provided empirical support for this notion, indicating that West German chess players, who had consistent access to chess computers during the 1980s, exhibited greater improvement over this period compared to their Soviet counterparts, who lacked similar access to these digital training aids.

Initially, the impact of chess computers on top players was limited. Early models, available commercially from 1977, were basic, offering only expert‐level play and little value to elite players. The 1990s changed this landscape as PCs became household items, capable of running increasingly powerful chess software. These engines, evolving in algorithms and computing strength, became indispensable to even the most skilled players. By the late 1990s, they rivalled the world's top players, and by the early 2000s, top players reported their inability to outmatch the premier engines running on standard PCs (Kasparov, 2010).

The arrival of the internet marked another significant transformation in the chess world around the same time. Previously, knowledge was transmitted via books or coaching, with electronic game records, available as chess databases, aiding training in the 1980s. However, PCs and the internet made this information widely accessible, breaking down traditional barriers. This change is reflected in the rise of Magnus Carlsen, the world's top player from Norway, a country with a limited chess history. Geographic constraints and the need for access to strong opponents or extensive chess libraries were eliminated. Now, with just a few clicks, players could access a wealth of chess games and analysis tools, revolutionizing learning and improvement in chess across the globe.

Evidence of AI's influence on elite chess performance in the 1990s can be observed through the decreasing age at which players achieve the grandmaster (GM) title, which is the highest level of skill in chess, awarded solely on performance, and generally very rarely achieved. Attaining the GM title typically requires prolonged, intensive practice and is closely connected to knowledge resources such as available coaching or books (Gobet, 2018). The record set by the legendary Bobby Fischer, who attained the grandmaster status at age 15 and 6 months in 1958, stood unchallenged for over three decades, until 1991, when Judith Polgar, a prodigious talent and a trailblazer in breaking gender barriers in chess, claimed the title at an even younger age (15 years and 4 months). Subsequently, throughout the 1990s, Polgar's record was surpassed nearly twenty times. By 2002, the record had been lowered to 12 years and 7 months, almost full 3 years younger than Fischer and Polgar, with the current record now standing at 12 years and 4 months (Mishra, 2022).

This rapid progression in the achievement of grandmaster status can be attributed to the combined impact of strong chess engines, widespread PC availability, and the proliferation of the internet, all of which significantly accelerated the accumulation of chess knowledge. More formal evidence supporting this trend was presented by Strittmatter et al. (2020), who demonstrated that the quality of play among world chess champions (and their opponents) has steadily improved over time, with a marked acceleration observed in the mid to late 1990s. This period of intensified progress aligns with the era of enhanced accessibility to advanced AI‐driven chess tools and resources.

The newest AI development in the late 2010s sent shockwaves through the (Go and) chess game communities. The new learning mechanisms based on deep learning and convoluted neural networks were clearly a different beast from the previous rather domain‐specific mechanisms relying on computation power mostly. Not only they could master complex domains such as Go, chess, and other games from the scratch with or no rules given, they were also markedly better than their predecessors. Initially, access to the groundbreaking self‐playing engine AlphaZero was limited, and even the games it played were scarce. In December 2017, only 10 games were released to the public, and it wasn't until a year later that an additional 200 games were made available. January 2019 saw the publication of a book containing many more games and detailed analyses of targeted experiments with AlphaZero (Sadler & Regan, 2019). Furthermore, in the preceding year, the open‐source engine LeelaChessZero brought the deep learning technology of AlphaZero within reach of chess practitioners. Soon after in 2020, one of the leading open‐source engines, Stockfish, implemented some of the deep neural network mechanisms, regained its throne as the best engine. Systematic records of changes in performance are lacking, but top players like Magnus Carlsen have acknowledged analysing games played by AlphaZero (Nielsen, 2019).

Recent studies have, however, demonstrated the impact of deep learning techniques, like those behind AlphaZero, in another complex game, Go. Choi et al. (2023) showed that the quality of individual decisions in Go games remained relatively stable until the release of a free source engine based on deep learning technology in early 2017. Subsequently, there was an improvement of over half a percentage point within a year, eventually plateauing at a two percentage point enhancement in less than 2 years. Notably, younger players exhibited considerably more improvement than their older counterparts. In another study, Shin et al. (2021) provided evidence that AI was indeed the driving force behind this enhancement in a natural experiment. Korean Army Go players, who were deprived of access to AlphaZero's open‐source counterpart, did not show significant improvement compared to their pre‐access performance, unlike their peers who had access to this new technology. Lastly, Shin et al. (2023) found that an increase in novelty was positively associated with the quality of individual decisions following the advent of deep learning AI in Go. This suggests that the new AI technology enabled Go players to devise innovative, previously unexplored strategies, leading to improved gameplay.

### Using AI techniques to uncover quantitative change in elite human performance

The influence of AI on human chess performance can be primarily attributed to two major advancements: (1) the accessibility of knowledge via the internet, coupled with powerful PCs which allowed for rapid development of chess engines in the late 1990s, and (2) the advent of neural networks and deep learning technology in the late 2010s. While anecdotal evidence exists for both these developments affecting top‐level chess play–such as the age of the youngest grandmasters and personal accounts regarding AlphaZero–a more systematic analysis is warranted. The study by Strittmatter et al. (2020) explored only world champions up until 2014, and while the impact of deep learning has been documented in Go (Shin et al., 2023), similar evidence in chess remains elusive.

Here we undertake a comprehensive examination of the performance of the top 20 chess players from 1985 to 2021. Our analysis focuses on their micro‐decisions, specifically individual moves within games, and compares them to the recommendations of the best chess engines available, thus providing an objective measure of their performance. Additionally, we contrast this elite group with two other samples: the top 20 young players (under 20 years old) and the top 20 senior players (over 65 years old). Drawing on previous research (Choi et al., 2023), we hypothesize that both the top‐tier and junior players will have benefitted more substantially from advances in AI than their senior counterparts. This hypothesis forms the basis of our study, detailed in our pre‐registration document (https://osf.io/tg6um).

Importantly, we adopt AI techniques analogous to those underpinning renowned chess engines like AlphaZero and Deep Blue to ascertain whether the shifts in performance, post‐technological advancements, are not merely quantitatively but qualitatively different, indicating a substantial deviation from previous levels of play. We use Multiple Change Points (MCP) analysis (Graf et al., 2023), a sophisticated statistical approach that excels in identifying significant departures from established trends (Raftery & Akman, 1986). In that sense, MCP mirrors the pattern recognition capability and dynamic adjustability based on given data central to the AI mechanisms behind chess engines.

## METHODS

### Sample

We provide all our data and the code necessary for the reported analyses at: https://osf.io/4k52p.

Pre‐registration can be found at: https://osf.io/tg6um. We focus on three groups: (1) top 20 players regardless of their age, (2) top 20 junior (under 20) players, and (3) top 20 seniors (above 65). The top players were chosen based on their Elo rating at the end of each year (September FIDE Elo list) for the top 20 in that year in their specific groups (top, junior, and senior). We focus on a span from 1985 to 2021 as this span includes both technological AI chess revolutions. The players were chosen based on their Elo ratings by using the official archive of the World Chess Federation, FIDE (https://ratings.fide.com/download.phtml). We included all games played by the chosen players in individual years from 1985 until 2021 from the commercially available ‘Mega database 2023’ with almost 10 million games from ChessBase (https://en.chessbase.com). Only tournament games under normal conditions, that is, not rapid, speed, or blitz chess, were included. These games were then analysed for individual moves. We excluded the first ten moves as they tend to be a well‐known theory at this level, as well as moves beyond move 60 where the small number of pieces presents considerable challenge for humans (for a similar approach and justification, see Smerdon, 2022). Overall, we analysed over 11.6 million individual decisions (half of them from the top 20 best players, 20 best juniors, and 20 best seniors, the other half coming from their opponents). If some of the top players, elite, junior, or senior, were not active in that particular year, that player had missing values.

### Measures

The quality of decision‐making will be measured by individual moves, more specifically, its quality as compared to the strongest chess engine, Stockfish. For comparison, the best human chess player, Magnus Carlsen, has an Elo rating of 2851 (the mean is around 1500, with a standard deviation of approximately 300–350 Elo points, see Vaci & Bilalić, 2017). The version of Stockfish 16 (with depth set at 15) we have used here has an Elo of over 3550 (https://stockfishchess.org/about/). This 700‐point rating difference corresponds to a win probability of about 99.9% for the open‐source engine. This means that Carlsen would have to play about 1000 games against Stockfish to have a chance of winning one game.

We will pit every move of every game for a player against the engine benchmark. Normally, the ‘centipawns’ deviation from the optimal move is obtained, indicating how much worse the chosen human move is, measured in hundredths of a pawn, from the engine move. The main problem with the use of centipawns is their heavy dependence on context. Losing 200 centipawns (around two pawns) in a balanced position is a significant blunder, but the same loss in centipawns in a situation where the game is already decided makes little difference and is irrelevant. There are ways around this problem, most notably by restricting the analysis to balanced, not already clearly decided, game portions (Backus et al., 2023; Guid & Bratko, 2007, 2011). Here, we adapt the approach currently favoured by the chess community, where the quality of play is expressed by accuracy for a single game. This essentially represents the deviation from the best decision (as benchmarked by a chess engine) or the percentage of optimal to good moves by the engine –100% means that all the chosen moves were those favoured by the engine. This approach avoids paradoxical situations where ‘mistakes’ of several hundred centipawns still lead to a won position because the advantage was already extremely high. We provide the code for calculating (an individual game's) accuracy based on chess engine evaluations in the online Data S1 (oSI). A less technical explanation can be found here: https://lichess.org/page/accuracy.

Besides accuracy, we provide three other measures of decision quality–aforementioned centipawns, the percentage of optimal move (the move that matches the move chosen by the computer in the same situation), and the percentage of win change compared to the engine move (how less likely in % is to win after the chosen move compared to the move chosen by the engine). Additionally, we also use centipawns to classify the errors players made within individual games. We are interested in three types of errors: (1) inaccuracies, which worsen the situation by more than 50 centipawns but less than 100; (2) mistakes, which lead to an evaluation of more than 100 centipawns worse than the optimal move but less than 200; and (3) blunders, which represent errors of a magnitude of 200 or more centipawns. The idea is to supplement the main accuracy measure with measures of another kind, which could additionally capture the variability of play.

Finally, we used the Swedish Computer Chess Association list to obtain the estimated Elo score for the best available chess engine/computer for each year between 1985 and 2021 (https://ssdf.bosjo.net/list.htm). We excluded from the computer list the computers which were not publicly available, such as Deep Blue or AlphaZeroChess, as none of the players could practice with them (and potentially improve).

### Analysis

We first average the accuracy scores for individual players for a given year across all the games in the given year. We do the same with the inaccuracies, mistakes, and blunders. All cases the number represents the average of the players in the given year across all games (per game, so to say).

We then correlated the Elo rating of the best AI with the averages of the dependent variables (e.g. accuracy, optimal move, centipawns, win percentage, inaccuracies, mistakes, and blunders) for each of the three groups over the same years. This will allow to estimate whether the quality of decisions improves similar as the AI's strength improved through the last 40 years.

In the next step, we applied Generalized Additive Models (GAMs) to analyse the results. GAMs were selected because they are well‐suited for capturing non‐linear relationships, using a data‐driven approach with non‐linear mixed‐effects regression (Wood, 2017). These models generate smooth curves for different groups, allowing for the assessment of group differences (see Graf et al., 2023; Vaci et al., 2019).

However, our goal here is not only to answer the hypothesis using classical methods employed in psychological research but also to illustrate how AI approaches, particularly machine learning, can be used to answer the same questions. We use change point analysis, which allows for the identification of abrupt changes in a sequence through significant deviations from established trends (Raftery & Akman, 1986). This differs from conventional linear regression analysis like ANOVA, Hierarchical Linear Modelling (HLM), or even non‐linear approaches such as GAMs because it specifically determines the exact moment of change in a function (Graf et al., 2023). It allows for the estimation of additional parameters, such as the intercept and slope before and after the change, and the exact time of the change. This is in contrast with other commonly used analysis for longitudinal data, HLM, which struggles to pinpoint the exact point when the performance curve changes (Gula et al., 2021; Long et al., 2024; Simonsohn, 2018).

The change point methodology is especially useful for identifying patterns of increase, where multiple change points might indicate underlying qualitative and quantitative shifts in human performance.

In this context, we employ the Multiple Change Points (MCP) package (Lindeløv, 2020), which estimates change points in data using Bayesian methods. It uses Markov Chain Monte Carlo (MCMC) sampling to infer posterior distributions for the locations of change points, according to user‐defined models. In a typical Bayesian approach, it combines prior knowledge with observed data and provides probabilistic estimates of change locations along with their associated uncertainties. This enables precise identification of change points and an assessment of their plausibility based on the data.

The MCP package also offers a way for evaluating both the plausibility of both individual parameters and the differences between models. The individual parameters are evaluated through the diagnostics of the MCMC sampling with Gelman‐Rubin convergence indicators (Gelman & Rubin, 1992). The difference between models is conducted by comparing models' predictive performance, considering both fit and complexity. Here we employ Leave‐One‐Out Cross‐Validation (LOO‐CV) for model validation (Shao, 1993). In LOO‐CV, each data point is sequentially excluded; the model is then retrained on the remaining data, and the prediction error for the excluded point is computed by averaging over all iterations. The resulting metric, the Estimated Log‐Predictive Density (ELPD), reflects the model's predictive power, indicating the model's credibility in accounting for out‐of‐sample data. When comparing two models, we consider the difference in their ELPDs, which includes the standard error (ELPD‐SE). The ratio of the ELPD difference to the ELPD‐SE serves as a Z‐score analogue: a ratio greater than 1.96 suggests a 95% probability that one model has superior predictive accuracy over another. Normally, one considers the difference important when the ratio is above two, which essentially means that the 2SE credible intervals around the difference do not encompass zero (Sivula et al., 2020). For the sake of readability, we will mostly present only the ratio. The ELPD for differences and its SE which are necessary for drawing credible intervals can be found in the oSI.

The chosen analytical approach here shares several similarities with the workings of chess engines. Like machine learning algorithms, MCP identifies patterns in chess performance data, revealing significant shifts akin to pattern recognition in AI. It adapts its model based on the data, much like machine learning algorithms adjust their parameters, ensuring precise identification of change points. Furthermore, MCP's Bayesian approach resonates with machine learning's principle of evolving understanding through new data, making it a potent tool for analysing complex patterns like those in chess performance.

## RESULTS

### Descriptive statistics

**FIGURE 1:**
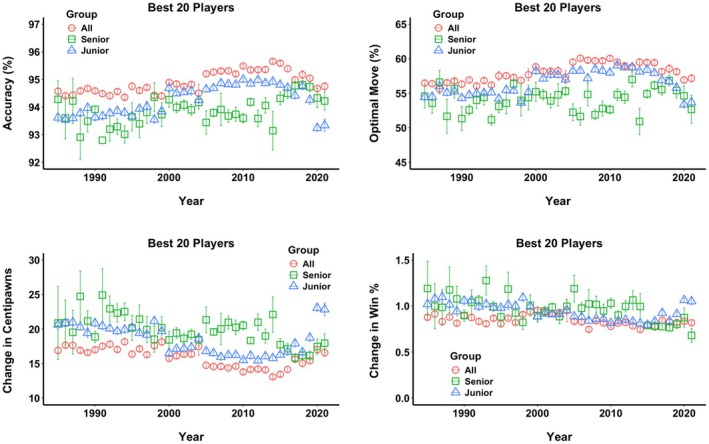
Quality of decisions of the best players from 1985 to 2021. Accuracy indicates how accurate the players were compared to the best AI; Optimal Move indicates the percentage of times the players chose the identical move as the best AI; Centipawns represent the absolute difference in centipawns (100 centipawns equal one pawn) between the move chosen by the players and the move chosen by the best AI; the same difference can be expressed in how much less likely the player is to win the game (in %) by Change in Win %. The larger the numbers in Accuracy and Optimal Move, the better the quality of decisions. For the Change in Centipawns and Change in Win %, the opposite is true, as these measures indicate the distance from the optimal move chosen by the best AI. All (red circles) represent the top 20 players, Junior represents the top 20 juniors (under 20 years of age, blue triangles), and Seniors are the top 20 seniors (over 65 years of age, green squares) if they played in that year. Error bars represent +/− 1 SE. [Correction added on 3 April 2025, after first online publication: Figures 1 and 2 have been updated in this version.]

Figure 1 presents the four indicators of decision‐making quality for the top players, juniors, and seniors from 1985 to 2021. In the first two indicators, Accuracy and Optimal Moves, the quality increases, indicating better overall decision‐making across time. In the second set of predictors, Change in Centipawns and Change in Win %, the trend is the same, but this time smaller values indicate better decision‐making as the differences between the best engine move and the moves chosen by players become smaller over time. The top players (red circles) demonstrate the highest accuracy, followed by juniors (blue triangles), while the seniors (green squares) have the lowest decision‐making quality.

There are a few trends regarding the development of decision‐making indicators in the three groups across the years. Top players tend to improve consistently over time, reaching peaks in the mid‐2000s and mid‐2010s. Juniors demonstrate a big jump in quality in the late 1990s, which is followed by a small but consistent increase in quality. The seniors have the most variation of all groups, reflecting the fact that many of the top 20 senior players did not play and consequently were not included in the analysis. Finally, in the top and junior players, there is a trend of worse decision‐making in the last 2 years of the analysed period, 2020 and 2021.

**FIGURE 2:**
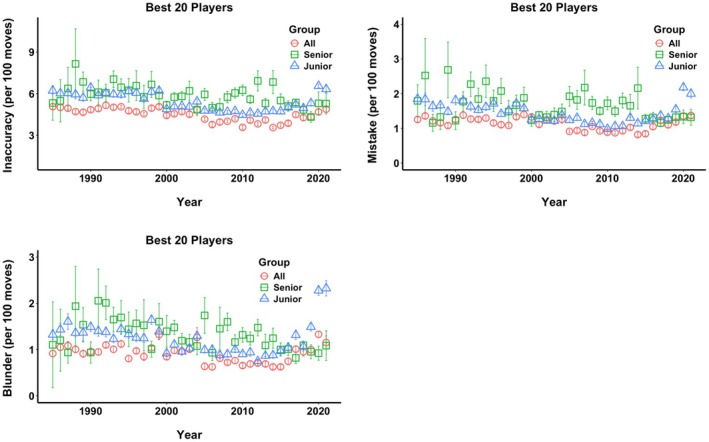
Errors made by the best players from 1985 to 2021. Average number of inaccuracies (more than 50 centipawns difference between the engine and the player's decision), mistakes (more than 100 centipawns), and blunders (more than 200 centipawns) per 100 moves for the top 20 players (All, red circles), top 20 under 20‐year‐olds (Junior, blue triangles), and the best 20 over 65‐year‐olds (Senior, green squares). Error bars represent +/− 1 SE.

Figure 2 illustrates the various types of errors committed by top players, top juniors, and top seniors between 1985 and 2021. We find the same patterns as with the quality of decisions–the top players made the fewest mistakes, followed by the best juniors, while the best seniors committed the most errors over the time period. We also find the same patterns when it comes to trends, where the top players made fewer and fewer errors, culminating in the mid‐2010s, while the best young players reduced the number of errors dramatically in the late 1990s. Top seniors did not exhibit noticeable changes in errors over time but did reduce their blunders.

### Impact of AI on performance–correlative analysis

**FIGURE 3:**
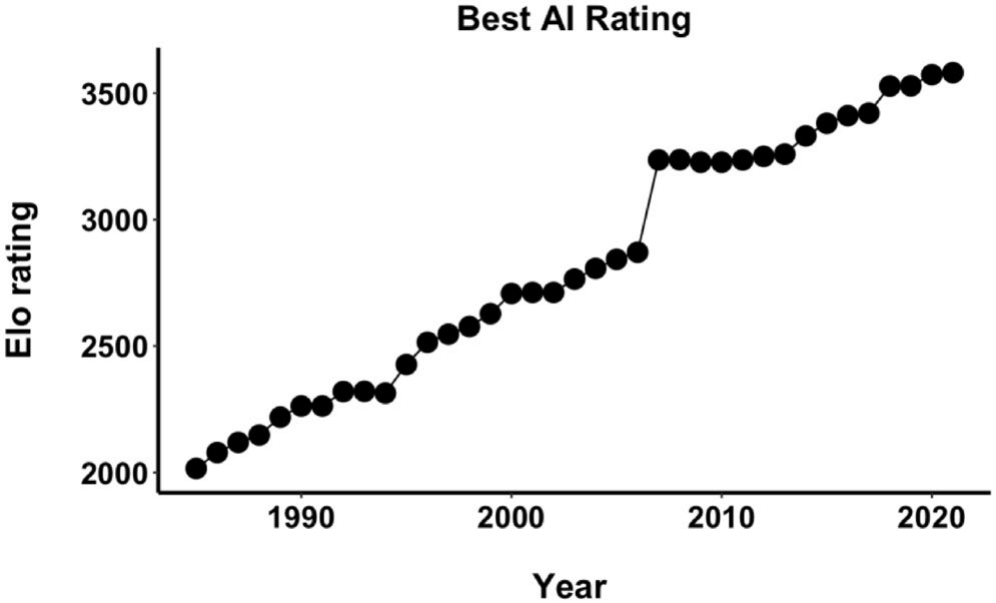
Elo rating of the best available chess AI (engine or computer) from 1985 to 2021. Deep Blue and AlphaZeroChess were excluded from the list, as they were not available to players. For comparison, the best human players had Elo ratings ranging from 2720 in 1985 (Garry Kasparov) to 2872 in 2013 and 2019 (Magnus Carlsen). The best AI matched these marks in 2002 (2712 Elo) and 2006 (2872 Elo).

We then check how these trends correspond with the AI improvements over the years. Figure 3 shows that AI improvements were particularly rapid until the early and mid‐2000s, reaching the level of the best human players. The mid‐2000s also saw a jump when the best AIs became too good even for the very best chess players. This jump in strength was followed by a relative stagnation for a few years, until the mid‐2010s, when there were sustained and constant small improvements.

Table 1 demonstrates that AI improvements coincided with the improvement in the quality of play among the top 20 players. The (Pearson's) correlation was highly positive for quality measures such as accuracy and optimal move share and negative for inaccuracies and mistakes, indicating overall better play with fewer errors. The correlation was particularly high in the very best players (All), somewhat weaker in junior players, and the weakest in senior players. However, when we formally compared the coefficients (see Section 1, in the SM), only the correlation between AI and optimal move share for all (.75) was significantly higher than the same correlation among the seniors (.18).

**TABLE 1:** Association between the strength of the best AI and the quality of decisions of the best player for the period between 1985 and 2021.

Variable	Age group	Pearson's r
Accuracy	All	0.75
Accuracy	Senior	0.52
Accuracy	Junior	0.57
Optimal Move	All	0.7
Optimal Move	Senior	0.18
Optimal Move	Junior	0.49
CentiPawns	All	−0.71
CentiPawns	Senior	−0.62
CentiPawns	Junior	−0.49
Win %	All	−0.47
Win %	Senior	−0.57
Win %	Junior	−0.65
Inaccuracy	All	−0.69
Inaccuracy	Senior	−0.49
Inaccuracy	Junior	−0.57
Mistake	All	−0.44
Mistake	Senior	−0.38
Mistake	Junior	−0.41
Blunder	All	−0.29
Blunder	Senior	−0.45
Blunder	Junior	−0.14

*Note*: All *r* significant at *p* < .05, except for Optimal Move Senior, and Blunder for All and Junior.

### Generalized additive model (GAM) analysis

In the next step, we formally describe the trends in the quality of decisions across groups over time by using modelling data for non‐linear trends with GAM. GAM allows us to estimate the trends over the years for each of the groups while capturing sudden changes such as improvements (Graf et al., 2023; Vaci et al., 2019). In all instances, we use models where both years (from 1985 to 2021) and age groups (All, Junior, and Senior) were modelled non‐linearly (using a smooth term). In addition, we also checked whether there is an interaction between years and age groups.

**FIGURE 4:**
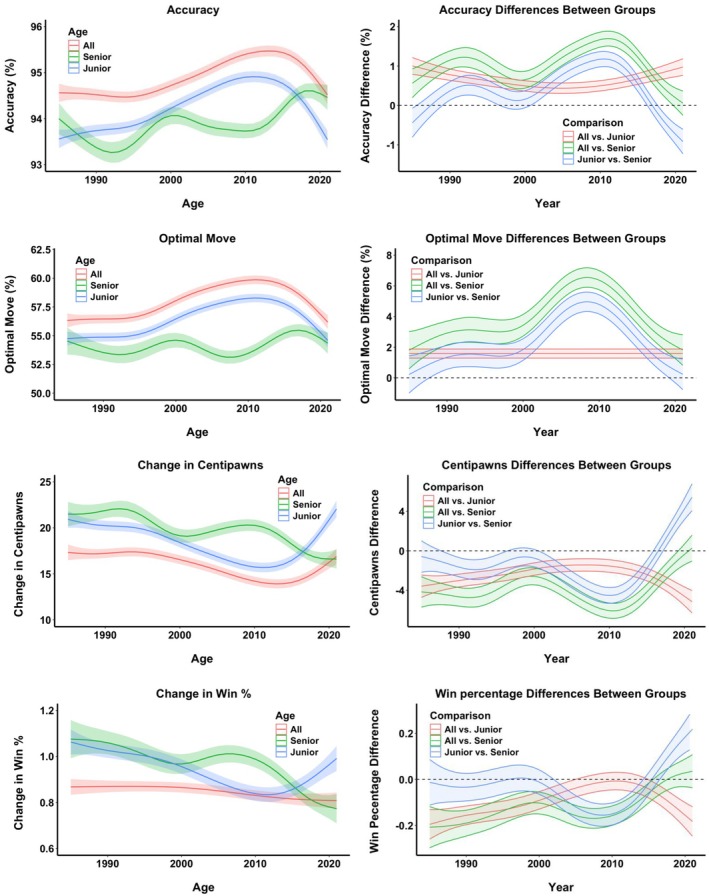
Modelling quality of decisions of the best players between 1985 and 2021. The left panel figures present non‐linear (Generalized Additive Models–GAMs) models for the best overall 20 players (All, red line), the best 20 under‐20‐year‐old players (Junior, blue line), and the best over‐65‐year‐old players (Senior, green line) for the period between 1985 and 2021 for the measures of quality of decisions (Accuracy–how precise they played, Optimal Move–how often they matched the best AI choice, Change in Centipawns–how far the chosen move was in centipawns from the best AI move, Change in Win %–how much less likely the player's chosen move was to win the game compared to the AI move). Error bars are shaded areas around the mean and represent 95% CI. The right panel plots the differences between the best overall players and juniors (red line), the best overall players and seniors (green line), and between juniors and seniors (blue line) for each of the quality of decision measures across all years. Error bars are shaded areas around the mean difference and represent 95% CI–the differences are significant if the CIs do not encompass zero, which is indicated by the dashed black line.

The models for each of the dependent variables are graphically presented in Figure 4 (left panel). In all instances, year and age group differences were significant, as well as the interaction between them. In other words, there were significant changes over the years, the age groups significantly differed from each other, and the differences between groups varied across the years (for more details, including specific significance tests, see the SM, Section 2). As can be seen in Figure 4 (left panels), all three groups mostly improved their decision quality across the years. The top players were making better decisions than the best young and old players, while the best junior players were mostly better than the best senior players.

The best players maintained a consistent quality of decisions from 1985 until the mid‐ and late 1990s, when both their accuracy and percentage of optimal moves began to increase. This improvement continued until the early 2010s, when it plateaued, and then, towards the very end of the 2010s, there was a decline. The best young players followed a similar trajectory, with the slight difference that their improvement was already visible before the late 1990s, and then, in the late 1990s, they saw significant progress. This improvement lasted until the early 2010s, followed by a reversal towards the end of the decade. In contrast, the best seniors experienced some valleys and peaks before the 2010s, most likely due to the sparse dataset (as several of the best seniors did not necessarily play every year). However, towards the end of the period, from the mid‐2010s until 2021, the quality of their decisions improved.

GAM also enables us to formally test for differences between groups across all time periods. Figure 4 (right panels) plots the differences between pairs of age groups, such as when we compare top players with junior players (red line in Figure 4, right panel), top players with senior players (green line), and junior players with senior players (blue line) between 1985 and 1991. Accompanying the differences are estimated 95% confidence intervals (CI) of the standard error of the differences (shaded areas). If these CIs do not encompass 0 (dashed black line in Figure 4, right panels), one can consider that the difference at that particular time point is significant. Consequently, we can see that for most of the time and in all quality of decision measures, the top players are significantly better at decision‐making than the best junior and especially senior players. The same can be said of junior players over senior players, with the notable exception of the last few years when the senior players have significantly better indicators.

The magnitude of these differences, however, provides clues about the patterns of improvement. For example, we can see that the top players hold a relatively constant edge in the quality of decisions over the best young players, but the advantage becomes smaller and smaller in the 2000s. Similarly, the senior players mostly make inferior decisions compared to the best overall and junior players, but towards the very end of the time period, not only does their performance improve, but the performance of the best junior players decreases, resulting in significantly better performance from the best senior players and no significant difference with the performance of the overall best players.

**FIGURE 5:**
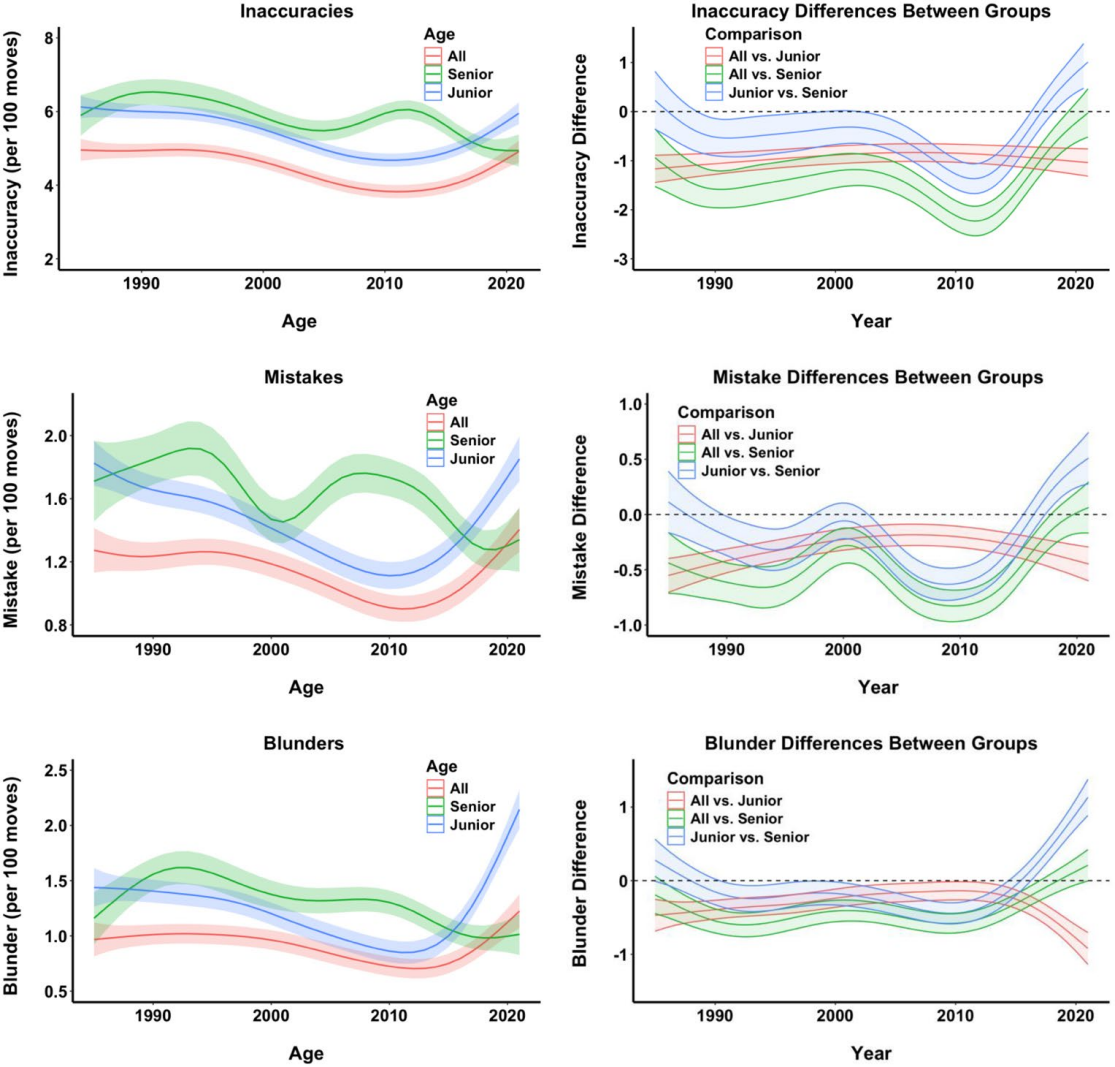
Modelling errors in decision of the best players between 1985 and 2021. The left panel figures present non‐linear (Generalized Additive Models–GAMs) models for the best overall 20 players (All, red line), the best 20 under‐20‐year‐old players (Junior, blue line), and the best over‐65‐year‐old players (Senior, green line) for the period between 1985 and 2021 for the measures of errors in decision‐making (Inaccuracy–how many times per 100 moves the chosen move was worse than 50 centipawns/half a pawn compared to the best AI move; Mistake–how many times per 100 moves the chosen move was worse than 100 centipawns/a pawn compared to the best AI move; Blunder–how many times per 100 moves the chosen move was worse than 200 centipawns/two pawns compared to the best AI move). Error bars are shaded areas around the mean and represent 95% CI. The right panel plots the differences between the best overall players and juniors (red line), the best overall players and seniors (green line), and between juniors and seniors (blue line) for each of the error measures in decisions across all years. Error bars are shaded areas around the mean difference and represent 95% CI–the differences are significant if the CIs do not encompass zero, which is indicated by the dashed black line.

We use the same GAM models for the errors in decision‐making (see SM, Section 2). Figure 5 (left panel) demonstrates that all groups make fewer errors over time, with the top players making the fewest, followed by juniors and seniors. The right panel of Figure 5 demonstrates that formal tests confirm this observation. We can again see that the advantage of the best players over the junior players becomes smaller over time, until the end of the period, when it is again large due to a considerable drop in performance among the junior players. This same drop towards the end is partly responsible for the significantly better performance of the seniors compared to the junior players. Mostly, however, it was the improved performance of the best senior players, who towards the end of the examined period made fewer and fewer errors.

### Multiple change points (MCP) analysis

We now turn to the formal analysis of identifying change points using the Multiple Change Points (MCP). Initially, we fit a model under the assumption of no change–essentially, a linear model–which serves as a baseline for comparison with other models. Subsequently, we fit models that incorporate one, two, or three change points, which may or may not vary across the age groups (see Table 2). The models where there is no variation between age groups assume that all three groups have similar patterns or results, i.e., represent the same population. While these models may not be plausible in this context, as we expect differences between the age groups, they are useful for comparison. These models are compared based on their fit, namely how well they describe the data, and the best is then chosen and presented here.

**TABLE 2:** Summary of multiple change point (MCP) models applied.

Model number	Model expression	Description
Model 1	dep ~ 1	Overall mean (global intercept) without Year or Age Group effects
Model 2	dep ~ 1 + Year	Overall mean (global intercept) with a slope for the Year variable
Model 3	dep ~ 1 + Year, ~ 1	Two intercepts (sudden jump in performance) with a slope for the Year variable before the sudden jump
Model 4	dep ~ 1, ~ 1 + Year	Two intercepts (sudden jump in performance) with a slope for the Year variable after the sudden jump
Model 5	dep ~ 1, ~ 1	Two intercepts (sudden jump in performance)
Model 6	dep ~ 1, ~ 1, ~ 1	Three intercepts (two sudden jumps in performance)
Model 7	dep ~ 1 + sigma(1)	Intercept model allowing for variance changes in decision quality
Model 8	dep ~ 1, 1 + (1|AgeGroup) ~ 1	Two intercepts (sudden jump in performance), but time when jump occurs varies between Age groups
Model 9	dep ~ 1 + Year, 1 + (1|AgeGroup) ~ 1	Two intercepts (sudden jump in performance) with a slope for the Year variable before the sudden jump, but time when jump occurs varies between Age groups
Model 10	dep ~ 1, 1 + (1|AgeGroup) ~ 1 + Year	Two intercepts (sudden jump in performance) with a slope for the Year variable after the sudden jump, but time when jump happens and slope after varies for age groups
Model 11	dep ~ 1, 1 + (1|AgeGroup) ~1, 1 + (1|AgeGroup) ~ 1	Three intercepts (sudden jump in performance) with a slope for the Year variable after the sudden jump, but time when jumps happens and slope after varies for age groups

*Note*: ‘dep’ indicates varying dependent variables for the quality and errors of decisions, such as Accuracy, Optimal Move, or Mistakes or Blunders. ‘Year’ represents the time period, i.e., years between 1985 and 2021, while ‘Age Group’ represents All, Senior, and Junior top 20 players.

In the case of Accuracy and Optimal move (for other quality and error indicators, see SM Section 3), by far the best fitting model was the model with a single sudden jump which varies among the age groups, as well as the subsequent slope (Model 10 in Table 1). This model is markedly better than the next best fitting model, the same model but where the slope does not vary among groups; this is evidenced by the difference in ELPD, which is −40.3, with an ELPD‐SE of 9.4, resulting in a ratio of 4.3 (for the full LOO statistics, see SM Section 3).

**FIGURE 6:**
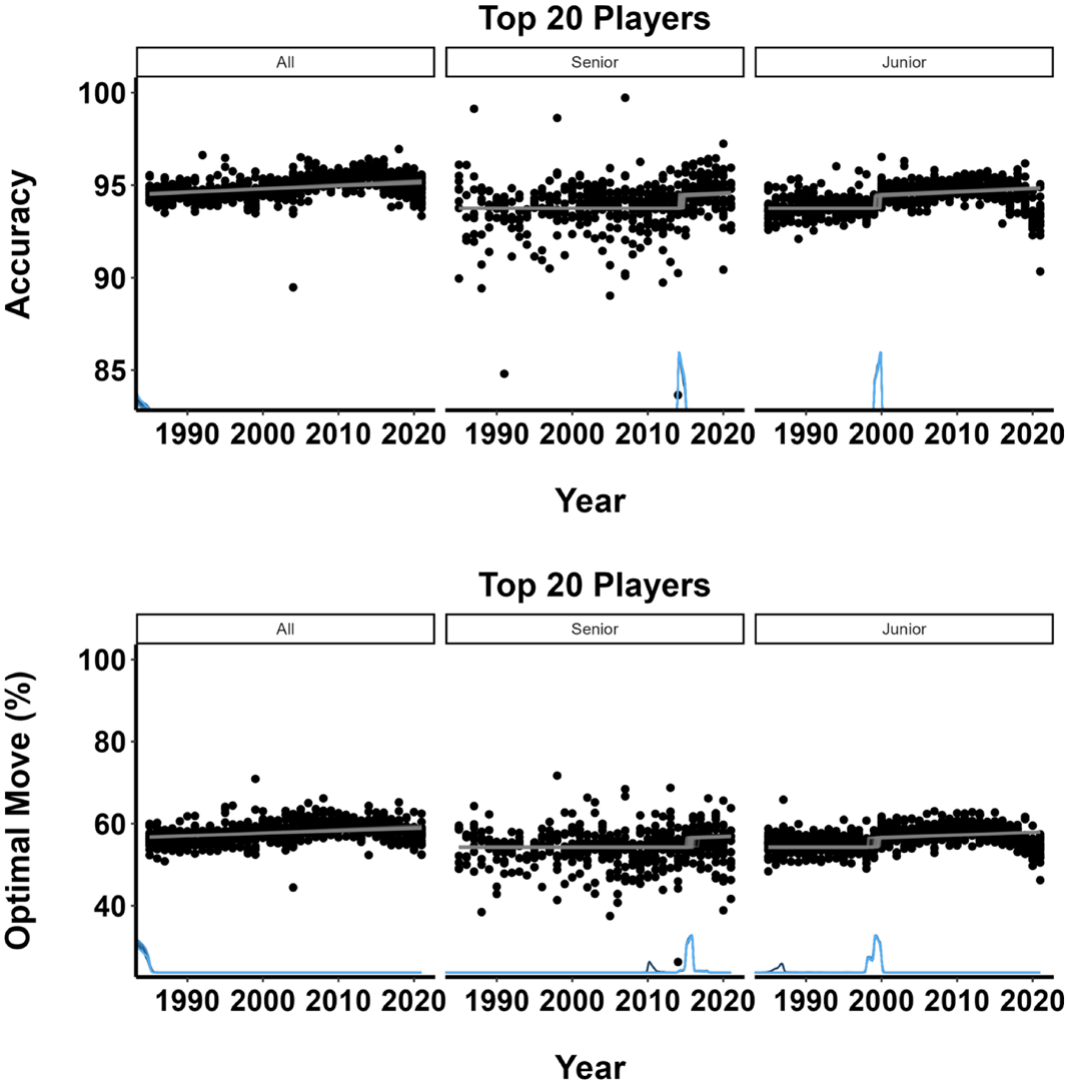
MCP analysis on the quality of decision in top chess players from 1985 to 2021. The models with two intercepts and two slopes, both varying across all age groups, are depicted. Black dots represent raw data (individual players). Grey lines represent randomly chosen draws from the estimated posteriors. Blue lines at the bottom present the posterior distributions of the estimated change points (one line for each of the four chains).

Figure 6 provides a graphical representation of the MCP analysis, depicting the accuracy and optimal move percentage for all three groups of players over the period from 1985 to 2021. The strength of the MCP analysis lies in its ability to precisely identify the timing of the change. The blue lines in Figure 6 depict the posterior distribution of the time at which the change occurs. For the top players, there is no significant change point for the accuracy, but the optimal moves percentage includes a single change point already at the beginning, around 1986 with over 90% of the estimates falling between 1985 and 1987. In contrast, the change for the best junior players was estimated to have occurred around 1999 in both accuracy and optimal move percentage, with the range between 1998 and 2000 encompassing nearly all the point change estimates (see the blue lines at the bottom of Figure 6). It is notable that the slope after the jump indicates consistent and constant increase in performance in the years after the jump. Finally, Figure 6 illustrates that the best seniors had a constant non‐improving performance until the mid‐2010 when they experienced a jump with ever‐increasing performance.

Despite GAM clearly identifying an improving quality of decisions since the mid‐1990s in the (overall) best players, it is notable that they did not have any significant break points. We also fitted individual MCP models only for the best players, which demonstrated that the significant breaks could be identified in particular in the mid‐2000s. However, none of the models was much better than the models when the (negative) change was for example in the late 2010s.

### Multiple change points (MCP) robustness analysis

We corroborate the MCP results by performing out‐of‐sample validation. Chess is particularly suitable for these kinds of robustness checks because the same players play games with both white and black pieces/sides. We leverage this feature of chess and first model all 11 MCP models on the data for the white side. We then use the results of all the models to predict the same model outcomes on the data when the same players play with the black pieces. Finally, we also perform a reverse out‐of‐sample validation where the black pieces were first modelled and then used to check how well they fit with the white pieces.

The best model was again the model with two intercepts (one jump) and a subsequent slope, where both the timing of the jump and the subsequent increase or decrease varied (Model 10 in Table 2). We provide Mean Square Error statistics for all the models and their predictions, from white to black, black to white, and the averages–see SM Section 4.

**FIGURE 7:**
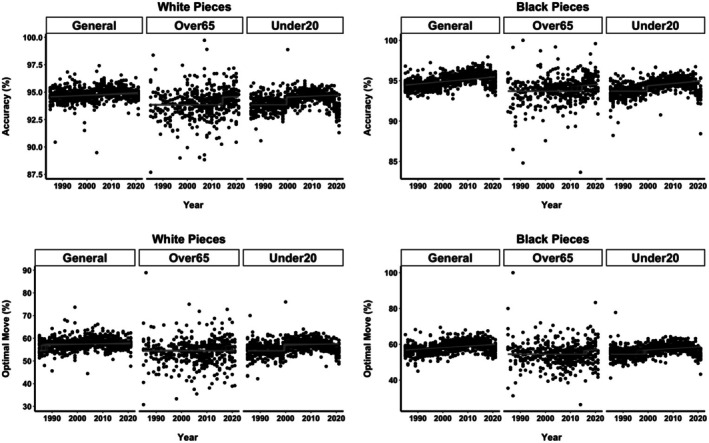
MCP robustness analysis on the quality of decisions in top chess players from 1985 to 2021. The models with two intercepts and two slopes, both varying across all age groups, are depicted for accuracy and optimal move share for the games played by white (left panels) and black pieces (right panel). Black dots represent raw data (individual players). Grey lines represent randomly chosen draws from the estimated posteriors. Blue lines at the bottom present the posterior distributions of the estimated change points (one line for each of the four chains).

Figure 7 presents a graphical representation of the best fitting model for the white and black pieces, for all three age groups, on the quality indicators of accuracy and optimal move share. These results confirm the findings on the whole sample, where the jumps for the overall best players are either none or early in the time period, while the sudden jumps occur in the late 1990s and mid‐2010s for juniors and seniors, respectively.

### Case study: Magnus Carlsen

Finally, we evaluated the performance of Magnus Carlsen, arguably the greatest chess player of all time and an early adopter of the new generation of deep learning‐based chess engines. Although Carlsen was beaten by Sergei Karjakin for the title of the youngest grandmaster by 10 months, he quickly ascended to the top 20 juniors in 2006, and by mid‐2011, he had become the world's number one rated chess player. He became the world chess championship in 2013 and successfully defended the title four times, most recently in 2021.

From July 2018 to October 2020, Carlsen remained unbeaten in 125 games, winning 42 and drawing 83 against top‐level opponents. This represents the record for the longest unbeaten streak in elite tournaments. What stands out during this period, particularly from 2018, is Carlsen's approach to the game. He began to clearly favour activity over material, often opting for sacrifices for long‐term advantages, a style typically avoided due to the uncertainty of outcomes (for a chess‐specific analysis, see GothamChess, 2021; Harding, 2019; Nielsen, 2019). Carlsen himself acknowledged the influence of AlphaZero, going so far as to call it his ‘hero’ in a press conference at Norway Chess 2019 in Stavanger (Nielsen, 2019).

**FIGURE 8:**
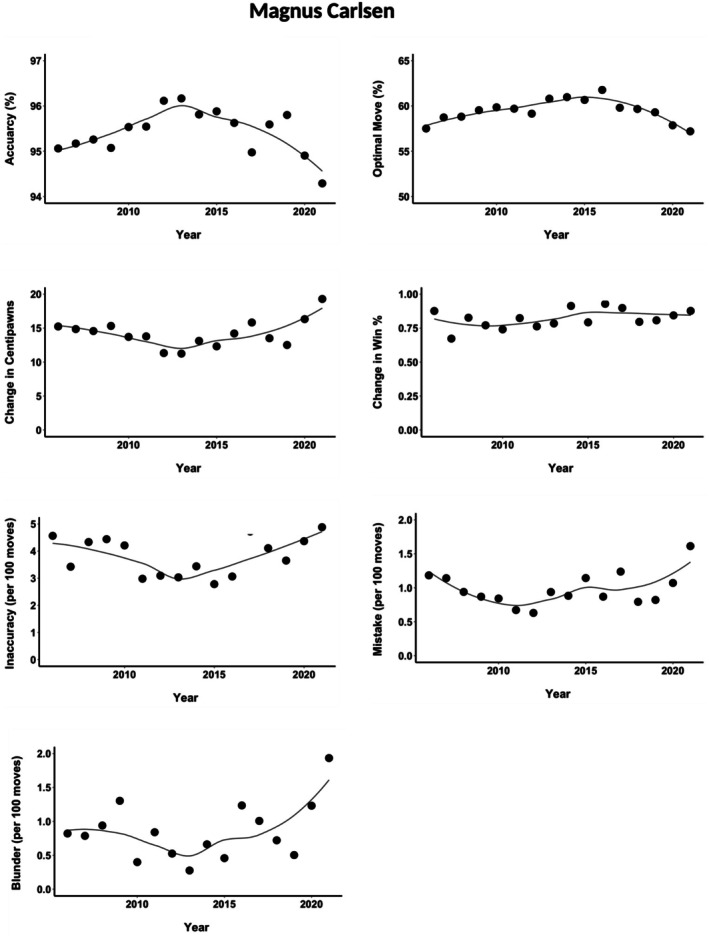
The quality of performance by the best world player (Magnus Carlsen) over the years. The fitted line is the non‐linear loess function.

This particular trend was not especially visible in Figure 8, which illustrates Carlsen's quality of play and the number of errors since 2006. In every aspect, Carlsen has enhanced his quality of play and consistency. This may not be surprising, as in 2006, he was not yet among the world's best players and had considerable room for improvement. However, his performance continued to improve even after 2011 when he reached the top ranking. There is a notable peak in his performance around 2012–2013. The performance in the record years 2018 and 2019 was among his best, but it did not quite reach the heights of the early 2010s (Figure 8).

## DISCUSSION

We set out to investigate the impact of the two AI chess revolutions on the performance of the very best chess players: the first one in the mid to late 1990s when the availability and power of PCs, combined with access to the internet, made chess computers strong enough for the best players to utilize them; and the second in the late 2010s when advances in deep learning and neural networks allowed for not only stronger chess computers but also a different kind of style of play. We find, however, that the best players improved the quality of their decision over the last four decades irrespective of age (see Figures 3 and 4). The improvement goes hand in hand with the improvement made in the chess AI domain, in particular for the very best players whose improvement rates were highly correlated with the improvement rates of the best available chess engines (see Table 1). Nevertheless, there were no discernable periods of time when the improvement rate was especially rapid in the case of the best players. Only the best young players improved significantly towards the end of the 1990s, indicating that they have benefited from the wide availability of PCs combined with access to chess knowledge through the internet. Finally, the new age of chess engines in the late 2010s does not seem to have impacted the quality of the best players, at least not yet.

### First AI chess revolution–mid to end of 1990s

Our study demonstrates that the quality of decisions among the best players steadily improved over the last 40 years. More remarkably, it was associated, particularly strongly among the very best group of players, with the steady improvements in chess AI. The best players did not start improving until the mid‐1990s, which might not be surprising. It is not unreasonable to assume that, despite all the advances in the strength of engines and the availability of knowledge in the 1980s and early 1990s, not much changed for the best players compared to the previous decade. Access to chess knowledge, including chess databases, was certainly different in the 1990s than in the 1980s, but the top players in the 1990s had probably acquired their knowledge in the pre‐internet era. The new access may have helped but likely not as much as it would have for other less knowledgeable players. Similarly, while chess engines in the early 1990s were improved over those in the 1980s, they were still not as strong as the best human players. As such, they were of limited use as sparring partners. Gaessler and Piezunka recently (Gaessler & Piezunka, 2023) found that access to chess computers has helped players improve their skills, but only for those who were weaker than the available chess computers. Only when the chess engine became good enough for use in training in the late 1990s and early 2000s, did the best players start improving.

On the other hand, the best young players improved steadily from the beginning (1985), and then suddenly around 1999, as confirmed by formal MCP analysis. The early improvements in the 1980s and early 1990s indicate that the young players, who were significantly weaker than the best players (the gap is around 200 Elo points, or 2/3 of SD), had more ‘opportunity’ to improve their performance (Pearson, 1902; Vaci et al., 2014) even when the best chess AI was of not much use. The young players, however, were in their formative years during the mid‐ and late 1990s, and the way they acquired knowledge would have been significant. This may have influenced their particularly sudden improvement in the late 1990s, when they also could benefit from the best available chess AI.

Our study only partly confirms the main conclusions from another study investigating a similar phenomenon. Strittmatter et al. (2020) found that individual decisions markedly improved in the mid to late 1990s, with over 8% in the share of optimal moves from mid‐1995–2010 (see Figure 2 in Strittmatter et al., 2020). This is a far cry from our results, where the top 20 players improved only by around 2% in the same time frame. One possible reason for this discrepancy is the differing designs. Our study included all the best players throughout the period, whereas Strittmatter and colleagues had a somewhat narrow focus on world champions (and their opponents in individual games) only.

### Second AI chess revolution–mid to end of 2010s

Unlike the first AI revolution, there is little evidence that the second AI revolution in the late 2010s played a role in affecting elite human performance. The players were not performing better, with the notable exception of the best senior players. However, even they started improving rapidly before the new chess AI became available in the mid‐2010s. It should also be noted that the average number of individual decisions in the early years was rather small for the senior players. However, from those couple of hundreds, it grew to almost one thousand decisions by the time the shift happened in the mid‐2010s. The shift may then simply reflect a more reliable dataset.

There are a number of possible reasons for the lack of effect of the new age chess AI on the best performers. Already by mid‐2000s, older chess engines had matched and surpassed any human player in terms of consistent performance throughout entire games (Elo of over 3000, while the best players had Elo over 2800, almost an SD gap). Consequently, even top players found these engines invaluable for training and preparation before the second AI chess revolution. While the new age engines further enhanced chess engine performance, the increase of less than 30 Elo points–arguably not really a significant leap over what was already a superior performance compared to the best humans (amounting to 700‐Elo gap or around two SDs)–may not have been large enough to impact top players' performance.

Similarly, the new style and ideas pioneered by the new chess engines may not lead immediately to visible improvement, at least on a consistent basis. We have seen that even in early adopters like Magnus Carlsen, who claimed to be greatly inspired by AlphaZeroChess, there were no visible objective improvements in the quality of decisions. As a matter of fact, Magnus Carlsen, as well as other top adult and youth players, experienced a considerable decline in the quality of decisions in the last two data points, 2020 and 2021, exactly when the AlphaZeroChess clone, LeelaChessZero, became available. This drop was not identified as a significant shift by the MCP analysis, but it is large enough to raise questions. We have taken great care to exclude the rapid and blitz tournaments, which could explain the sudden drop in performance. By that time, however, much shorter control times became prevalent. Having less time for making complex decisions may have negatively impacted the quality of those decisions. Another possible reason is the recent pandemic and the unusual tournament play from home, which was found to be a significant, if not to this extent, deteriorator of performance (Smerdon, 2022).

### Shortcomings and future directions

We are aware that our evidence for the impact of the first major AI chess revolution is largely circumstantial. We do observe the influence of AI on chess performance, particularly for the top juniors in the 1990s, and these findings are robust, as evidenced by our leave‐one‐out sample approach. However, it is undeniable that other factors might also be at play, and not only that AI advances were available at that particular point in time. Ideally, one would require a natural experiment (see Vaci et al., 2025) similar to the one described in Shin et al. (2021), which took advantage of the lack of access to new AI technologies and its impact on the improvement of Go players who were serving in the Korean army at the time of the new Go engine's release. A more feasible approach, however, might involve expanding the scope and sample size of the current study. For instance, one could test the assumption that only top players benefit from the new age chess engines by examining a broader spectrum of high‐level players. At a certain skill threshold, one might expect the benefits of these engines to diminish or disappear entirely. Likewise, the accessibility of knowledge in the 1990s may have aided junior players due to their youth, rather than their skill level. Broadening the focus to include players of similar skill but older age could help determine the validity of this assumption.

The AI's effects on the performance of the best chess players of all ages were statistically significant and reliable, but they were not substantial. These effects were certainly less pronounced than those observed in Go studies (Choi et al., 2023; Shin et al., 2021, 2023), where the impact of freely available engines based on AlphaZero was quite dramatic immediately following their release. There are potentially two mechanisms at play here. Firstly, the Go studies encompassed a broad spectrum of players and employed different measures of decision quality. It is possible that the inclusion of a wider range of player skill levels is significant.

Secondly, Go is inherently more complex than chess due to, among other things, larger search space. As a result, even the best Go players arguably had more to learn compared to chess players, where the engines had already achieved a very high level of play. For instance, the top Go computer before AlphaZero, CrazyStone, was rated at only 2000 Elo with the best human Go player having almost 4000 Elo. The AlphaZero pushed the level of play above 5000 Elo (Choi et al., 2023). In chess, the top engines were already rated much higher than the best human players (around 3050 vs. 2850 Elo). We are not aware of available accuracy measures for Go, but it is reasonable to assume that they would be lower than those achieved by the best chess players, some of whom have reached a 97% accuracy rate over the course of a year. This difference in game complexity and player learning potential may account for the more pronounced effects observed in the Go studies.

Finally, our study offers limited insight into the specific mechanisms behind the performance improvements of the best chess players in the era of advanced chess engines. We had hoped that analysing the number of errors might shed light on the underlying processes. While most elite groups reduced their errors, there were no clear patterns of sudden improvement, especially none directly linked to the two AI chess revolutions. Similarly, our case study did not provide new insights either. Future research could adopt a more systematic and quantitative method to explore how AI influences human performance in chess.

For instance, a commonly cited explanation for the enhanced performance of top players is the introduction of new ideas and inspiration. Investigating this in chess is challenging, but there are promising methods. One such approach is illustrated by Shin et al. (2023), who found that an increase in performance quality in Go was associated with novelty, that is introduction of previously unseen continuations. Similar research in chess could yield further insights.

Another approach could involve examining the cultural evolution of chess play and how it has been shaped by external factors. For example, Lappo and colleagues (2023) demonstrated how factors like familiarity, reputation, and personal success have influenced the decision‐making (move choices) of top players. They also showed how analysis with chess engines led to a deeper understanding of certain types of positions, influencing their frequency of use in practice. Such studies could provide a more fine‐grained understanding of the AI's impact on chess.

### Conclusions

Our study established an association between the performance improvements of the best chess players and advancements in chess AI. While the overall progress was gradual, the youngest top players nevertheless displayed accelerated gains during a key AI breakthrough. Our findings demonstrate that even the most elite performers can benefit from AI developments when the conditions are right–a conclusion that has remained elusive in recent research on AI's impact on human performance (Dell'Acqua et al., 2023; Noy & Zhang, 2023). Our results align well with the perspective (Kasparov, 2017; Mollick, 2024) that recent AI advancements offer a unique opportunity for collaboration, rather than competition, even at the pinnacle of chess–a game long regarded by the AI field as a quintessential example of human intelligence. This underscores the potential for AI to enhance human ability in complex tasks, rather than simply competing against or replacing human skills.

## AUTHOR CONTRIBUTIONS

**Merim Bilalic:** Conceptualization; data curation; formal analysis; visualization; writing – original draft; methodology; investigation; supervision; project administration; writing – review and editing. **Mario Graf:** Data curation; formal analysis; writing – review and editing. **Nemanja Vaci:** Conceptualization; data curation; formal analysis; visualization; writing – review and editing.

## CONFLICT OF INTEREST STATEMENT

The authors declare no conflict of interests.

## Supporting information


Data S1.

## Data Availability

Pre‐registration: https://osf.io/tg6um. Data and analyses: https://osf.io/4k52p
