# Supplementary material for: Computers and chess masters: The role of AI in transforming elite human performance
Source: Br J Psychol. 2024 Dec 5;117(2):585–609. doi: 10.1111/bjop.12750 (PMC13051028; doi:10.1111/bjop.12750)
Supplement: Supplementary file 1 — Data S1. [file BJOP-117-585-s001.pdf]

SUPPLEMENTARY MATERIAL

Computers and Chess Masters:

The Role of AI in Transforming Elite Human Performance

Preregistration can be found at:

https://osf.io/tg6um/?view_only=ad3847e45a4747e08453c35328f4de60

Online Supplemental Material (oSM) can be found at:

https://osf.io/4k52p/?view_only=9737a724f4ca4d20af785c4a8af54c8e

Section 1. Association between chess AI and chess quality of decision of the best player over time

We compared the correlations between AI performance and indicators of quality and error of decisions (such as accuracy, optimal share, change in CP, etc.) across three groups: All, Senior, and Junior. We used Fisher's Z transformation to compare the correlations between AI and the variables in each group. Here are the results:

Table SM1. Comparison of the correlations between AI performance and indicators of quality and error of decisions (p-values, two-tailed).

AI with	All_vs_Senior	All_vs_Junior	Senior_vs_Junior
Accuracy	0.09	0.19	0.74
Optimal Move	0.004	0.17	0.14
Centipawn	0.50	0.14	0.42
Win Percentage	0.56	0.28	0.62
Inaccuracies	0.19	0.42	0.62
Mistakes	0.74	0.88	0.86
Blunders	0.44	0.53	0.16

Section 2. GAM analysis

In the main text we provided the graphical presentation of the results. Here we provide the formal test of the variables (Year and Age Group) and their interactions.

Table SM2. GAM Results (from left to right, top to bottom: Accuracy, Optimal Move, Centipawns, Win %, Inaccuracies, Mistakes, and Blunders).

```
Formula:
Accuracy ~ s(Year) + Age + s(Year, by = Age)

Parametric coefficients:
            Estimate Std. Error t value Pr(>|t|)
(Intercept) 94.91953    0.02939 3229.84   <2e-16 ***
AgeOver65   -1.02470    0.04744  -21.60   <2e-16 ***
AgeUnder20  -0.64654    0.04150  -15.58   <2e-16 ***
---
Signif. codes:  0 ‘***’ 0.001 ‘**’ 0.01 ‘*’ 0.05 ‘.’ 0.1 ‘ ’ 1

Approximate significance of smooth terms:
                          edf Ref.df      F  p-value
s(Year)              6.267412      9 21.796  < 2e-16 ***
s(Year):AgeGeneral   0.005191      9  0.001   0.0153 *
s(Year):AgeOver65    5.683270      9 12.478  < 2e-16 ***
s(Year):AgeUnder20   2.402832      9  2.419 5.65e-06 ***
---
Signif. codes:  0 ‘***’ 0.001 ‘**’ 0.01 ‘*’ 0.05 ‘.’ 0.1 ‘ ’ 1

R-sq.(adj) =   0.34   Deviance explained = 34.6%
-REML = 1496.3  Scale est. = 0.62976   n = 1968
```

```
Formula:
OptimalMove_100 ~ s(Year) + Age + s(Year, by = Age)

Parametric coefficients:
            Estimate Std. Error t value Pr(>|t|)
(Intercept)  58.0873     0.1070  543.08   <2e-16 ***
AgeOver65    -3.9458     0.1728  -22.84   <2e-16 ***
AgeUnder20   -1.5846     0.1508  -10.51   <2e-16 ***
---
Signif. codes:  0 ‘***’ 0.001 ‘**’ 0.01 ‘*’ 0.05 ‘.’ 0.1 ‘ ’ 1

Approximate significance of smooth terms:
                           edf Ref.df     F p-value
s(Year)              5.827e+00      9 36.61  <2e-16 ***
s(Year):AgeGeneral   8.391e-05      9  0.00   0.648
s(Year):AgeOver65    4.835e+00      9 12.10  <2e-16 ***
s(Year):AgeUnder20   4.389e-03      9  0.00   0.542
---
Signif. codes:  0 ‘***’ 0.001 ‘**’ 0.01 ‘*’ 0.05 ‘.’ 0.1 ‘ ’ 1

R-sq.(adj) =  0.307   Deviance explained = 31.1%
-REML = 3076.5  Scale est. = 8.3733    n = 1968
```

```
Formula:
ChangeCP ~ s(Year) + Age + s(Year, by = Age)

Parametric coefficients:
            Estimate Std. Error t value Pr(>|t|)
(Intercept)  15.8383     0.1216   130.2   <2e-16 ***
AgeOver65     3.8868     0.1963    19.8   <2e-16 ***
AgeUnder20    2.4741     0.1718    14.4   <2e-16 ***
---
Signif. codes:  0 '***' 0.001 '**' 0.01 '*' 0.05 '.' 0.1 ' ' 1

Approximate significance of smooth terms:
                        edf Ref.df     F  p-value
s(Year)              4.7277      9 7.185  < 2e-16 ***
s(Year):AgeGeneral 0.8625      9 1.115 2.77e-07 ***
s(Year):AgeOver65  5.1108      9 9.009  < 2e-16 ***
s(Year):AgeUnder20 3.7706      9 6.071  < 2e-16 ***
---
Signif. codes:  0 '***' 0.001 '**' 0.01 '*' 0.05 '.' 0.1 ' ' 1

R-sq.(adj) =  0.308   Deviance explained = 31.4%
-REML = 3239.4  Scale est. = 10.788     n = 1968
```

```
Formula:
ChangeWinPer ~ s(Year) + Age + s(Year, by = Age)

Parametric coefficients:
            Estimate Std. Error t value Pr(>|t|)
(Intercept)  0.84525    0.00804 105.135  < 2e-16 ***
AgeOver65    0.11907    0.01297   9.181  < 2e-16 ***
AgeUnder20   0.09012    0.01136   7.933 3.56e-15 ***
---
Signif. codes:  0 '***' 0.001 '**' 0.01 '*' 0.05 '.' 0.1 ' ' 1

Approximate significance of smooth terms:
                         edf Ref.df     F  p-value
s(Year)            1.671e+00      9 1.262 0.000586 ***
s(Year):AgeGeneral 8.475e-05      9 0.000 0.064519 .
s(Year):AgeOver65  4.147e+00      9 5.051  < 2e-16 ***
s(Year):AgeUnder20 3.854e+00      9 4.783  < 2e-16 ***
---
Signif. codes:  0 '***' 0.001 '**' 0.01 '*' 0.05 '.' 0.1 ' ' 1

R-sq.(adj) =  0.127   Deviance explained = 13.2%
-REML = -105.29  Scale est. = 0.047197   n = 1968
```

```
Formula:
Inaccuracy_100 ~ s(Year) + Age + s(Year, by = Age)

Parametric coefficients:
            Estimate Std. Error t value Pr(>|t|)
(Intercept)  4.44409    0.04996   88.96   <2e-16 ***
AgeOver65    1.38489    0.08064   17.17   <2e-16 ***
AgeUnder20   0.94156    0.07052   13.35   <2e-16 ***
---
Signif. codes:  0 '***' 0.001 '**' 0.01 '*' 0.05 '.' 0.1 ' ' 1

Approximate significance of smooth terms:
                        edf Ref.df      F p-value
s(Year)            5.064435      9 12.999  <2e-16 ***
s(Year):AgeGeneral 0.000811      9  0.000  0.0400 *
s(Year):AgeOver65  5.160729      9  7.206  <2e-16 ***
s(Year):AgeUnder20 1.297371      9  0.443  0.0412 *
---
Signif. codes:  0 '***' 0.001 '**' 0.01 '*' 0.05 '.' 0.1 ' ' 1

R-sq.(adj) =  0.224   Deviance explained =   23%
-REML = 2140.8  Scale est. = 1.8214     n = 1968
```

```
Formula:
Mistake_100 ~ s(Year) + Age + s(Year, by = Age)

Parametric coefficients:
            Estimate Std. Error t value Pr(>|t|)
(Intercept)  1.12721    0.02284   49.35   <2e-16 ***
AgeOver65    0.50527    0.03686   13.71   <2e-16 ***
AgeUnder20   0.30318    0.03225    9.40   <2e-16 ***
---
Signif. codes:  0 '***' 0.001 '**' 0.01 '*' 0.05 '.' 0.1 ' ' 1

Approximate significance of smooth terms:
                         edf Ref.df     F  p-value
s(Year)            4.8837378      9 6.960  < 2e-16 ***
s(Year):AgeGeneral 0.0008359      9 0.000 0.004095 **
s(Year):AgeOver65  5.4148218      9 7.535  < 2e-16 ***
s(Year):AgeUnder20 2.0787645      9 1.614 0.000264 ***
---
Signif. codes:  0 '***' 0.001 '**' 0.01 '*' 0.05 '.' 0.1 ' ' 1

R-sq.(adj) =  0.167   Deviance explained = 17.3%
-REML = 1181.7  Scale est. = 0.38048    n = 1968
```

```
Formula:
Blunder_100 ~ s(Year) + Age + s(Year, by = Age)

Parametric coefficients:
            Estimate Std. Error t value Pr(>|t|)
(Intercept)  0.90644    0.02269  39.951   <2e-16 ***
AgeOver65    0.39355    0.03660  10.753   <2e-16 ***
AgeUnder20   0.31903    0.03205   9.955   <2e-16 ***
---
Signif. codes:  0 '***' 0.001 '**' 0.01 '*' 0.05 '.' 0.1 ' ' 1

Approximate significance of smooth terms:
                         edf Ref.df     F p-value
s(Year)            4.9634584      9 5.267  <2e-16 ***
s(Year):AgeGeneral 0.0008879      9 0.000   0.014 *
s(Year):AgeOver65  4.1210663      9 4.781  <2e-16 ***
s(Year):AgeUnder20 4.4652504      9 4.540  <2e-16 ***
---
Signif. codes:  0 '***' 0.001 '**' 0.01 '*' 0.05 '.' 0.1 ' ' 1

R-sq.(adj) =  0.171   Deviance explained = 17.8%
-REML = 1176.2  Scale est. = 0.37535    n = 1968
```

Section 3. MCP Analysis

Here we provide model fits and their comparisons for the presented models for Accuracy and Optimal Move.

Table SM3. MCP model comparisons (LOO) results for Accuracy.

Models	elpd_diff	se_diff	elpd_loo	se_elpd_loo	p_loo	se_p_loo	looic	se_looic
Model10	0.0	0.0	-2455.7	113.4	15.8	6.6	4911.4	226.7
Model8	-40.3	9.4	-2496.0	113.5	15.9	6.6	4991.9	227.0
Model11	-64.7	8.7	-2520.3	108.9	46.9	10.1	5040.7	217.8
Model5	-218.4	26.1	-2674.1	101.5	11.8	5.3	5348.1	203.0
Model4	-219.5	25.9	-2675.2	101.6	13.1	5.6	5350.5	203.3
Model9	-225.0	24.2	-2680.7	109.5	98.9	14.2	5361.3	219.1
Model3	-227.6	29.3	-2683.3	103.4	22.9	6.1	5366.7	206.8
Model6	-271.4	26.4	-2727.1	102.1	54.7	11.6	5454.3	204.3
Model1	-296.3	31.4	-2751.9	93.0	9.5	4.4	5503.9	186.1
Model7	-296.4	31.3	-2752.1	93.2	9.5	4.5	5504.1	186.3
Model2	-299.3	29.5	-2755.0	96.1	13.3	5.7	5509.9	192.1

Table SM4. MCP model comparisons (LOO) results for Optimal Move.

Models	elpd_diff	se_diff	elpd_loo	se_elpd_loo	p_loo	se_p_loo	looic	se_looic
Model10	0.0	0.0	-2455.7	113.5	15.8	6.7	4911.4	226.9
Model8	-40.3	9.3	-2496.0	113.4	16.2	6.7	4992.0	226.9
Model9	-86.0	19.7	-2541.7	108.4	25.0	6.6	5083.4	216.8
Model11	-190.2	14.1	-2645.9	110.0	149.9	14.6	5291.8	220.1
Model5	-218.5	26.1	-2674.1	101.5	11.7	5.3	5348.3	202.9
Model4	-219.3	26.0	-2674.9	101.5	12.8	5.4	5349.9	202.9
Model3	-229.7	29.6	-2685.4	103.2	22.2	6.1	5370.8	206.4
Model6	-287.4	28.3	-2743.1	97.6	35.2	8.4	5486.1	195.3
Model2	-289.1	29.2	-2744.8	96.4	10.6	5.0	5489.6	192.8
Model7	-296.1	31.5	-2751.8	93.0	9.2	4.3	5503.6	186.1
Model1	-296.3	31.4	-2752.0	93.1	9.4	4.5	5504.0	186.3

All graphs and statistics for other measures of quality and errors of decisions can be found in oSM.

Section 4. MCP Analysis – leave-one-out White to Black and Black to White robustness check

Here we provide a table with Mean Square Errors (MSE) for the prediction of all models for Accuracy and Optimal Move. In both instances, Model 10 has the smallest MSE.

Table SM5. Leave-one-out prediction fits for all models for Accuracy (from white pieces to black, from black pieces to white, and the average of both).

Model	MSE (White to Black)	MSE (Black to White)	Both
Model1	1.484	0.941	1.213
Model2	1.367	1.033	1.2
Model3	1.393	0.962	1.178
Model4	1.405	0.958	1.181
Model5	1.4	0.957	1.179
Model6	1.443	0.916	1.179
Model7	1.484	0.941	1.213
Model8	1.484	0.863	1.173
Model9	1.2	0.842	1.021
Model10	1.184	0.834	1.009
Model11	1.214	0.818	1.016

Table SM6. Leave-one-out prediction fits for all models for Optimal Move (from white pieces to black, from black pieces to white, and the average of both).

Model	MSE (White to Black)	MSE (Black to White)	Overall
Model1	1434.812	1477.992	1456.402
Model2	1434.311	1477.884	1456.097
Model3	1434.227	1477.732	1455.98
Model4	1434.397	1477.842	1456.119
Model5	1434.422	1477.818	1456.12
Model6	1434.451	1477.976	1456.213
Model7	1434.816	1478.01	1456.413
Model8	1433.599	1477.159	1455.379
Model9	1433.456	1477.45	1455.453
Model10	1433.461	1477.002	1455.231
Model11	1433.766	1477.32	1455.543

All graphs and statistics for other measures of quality and errors of decisions for white and black pieces, as well as their robustness check, can be found in oSM.

PREREGISTRATION CHECK

From Pawns to Neural Networks: Analyzing AI's Impact on Chess and Human Behavior

Preregistration can be found at:

https://osf.io/tg6um/?view_only=ad3847e45a4747e08453c35328f4de60

Description

Technology has always played a pivotal role in reshaping the way people live. From the inception of electricity, which brought light to homes and powered machinery, to the invention of personal computers, which revolutionised how we process information and communicate. Recently, technological advancements such as machine learning, large language modelling, and neural network-based vision systems have led to a paradigm shift in numerous sectors. From healthcare, where AI-driven diagnostic tools are enhancing accuracy, to finance, where algorithmic trading dominates the markets, the influence of these advancements is omnipresent.

This transformative wave, primarily driven by innovations in technology, is evident in the way people live and behave. It's commonly believed that such changes are largely beneficial, simplifying tasks and rendering them more efficient. However, gauging the full extent of technology's impact on human behaviour remains challenging. In this context, we will focus on the game of chess, an intellectual domain par excellence with a long tradition and available objective measures of performance. Chess provides an insightful example as it represents a field where technological advancements have had a clear and measurable impact.

We have two goals in mind. On the one hand, we want to demonstrate how new technologies, such as machine learning-based computer engines, have impacted the performance of the best chess players by analysing performance throughout history. This can be seen in the rise of computer-aided training methods, access to vast databases of historical games, and the ability to simulate games against virtual opponents of various skill levels. The use of AI in chess has not only elevated the performance of top players but also democratised access to high-level training resources, reshaping the way the game is played and learned at all levels.

On the other hand, we aim to utilise some of these transformative AI technologies in our own analysis. We seek to illustrate how the same machine learning algorithms that have catalysed improvements in one real-world domain can similarly offer insights in psychology. In doing so, we not only reinforce our understanding of AI's impact on an intellectual realm but also provide an example of its prospective applications in the field of psychology.

Study Information

Hypotheses

We expect an increase in quality of the decision making over time in all three groups (H1)

We also expect that the increase in quality of the decision making over time will correlate with the strength of the chess engines over time (H2).

We assume that the presence and availability of machine learning based chess engines will improve the quality of the decision making (H3). We don't however believe that this effect will be strong because a) it has only recently occurred (around 2018) and may need more time and b) the classical chess engines were already very strong and neural networks add only a small amount of strength (Stockfish integration with Lc0 in 2020).

Finally, we expect that the junior and general sample of players will benefit more than senior players because of their early adaptation of technology (H4).

Design Plan

Study type

Observational Study - Data is collected from study subjects that are not randomly assigned to a treatment. This includes surveys, "natural experiments," and regression discontinuity designs.

Blinding

No blinding is involved in this study.

Is there any additional blinding in this study?

No response

Study design

The goal is to investigate yearly changes in the quality of decision making of the best chess partitioners throughout recent history.

Sample.

We will focus on three groups:

1) top 20 players regardless of their age,
2) top 20 junior (under 20) players, and
3) top 20 seniors (above 65)

The top players will be chosen based on their Elo rating for each year and we will use lists of best players for each year from 1985 to 2021.

We will identify all games of players on the lists in ChessBase Big Database 202. Every move of every game for a player will be compared against the engine benchmark, and following measures will be obtained:

1) Individual games accuracy (see https://lichess.org/page/accuracy)
2) Share of optimal moves (i.e. moves chosen by the engine too) in individual games
3) Centipawns changes for each move in individual games.

These measures will be averaged for each player, for the year of interest. In other words, all moves and games in a certain year of a certain players will be averaged to get a single value for 1) average game accuracy in that year, 2) average share of optimal moves, and 3) average of centipawns for all moves, and 4) count of inaccuracies, mistakes, and blunders.

Independent Measures: In addition to the already mentioned the cohort variable (i.e. junior, top, and senior players), we will have two other factors.

The first will be the highest Elo rating of chess engine in particular year, starting from 1985, the same year we have the data for the dependent measures.

The second factor will take into account technological advances throughout the years, and will include the following milestones : a) readily available personal computers (PCs) with strong chess engines and available database of games (around the mid-1990s to early 2000) , and b) readily available neural network chess engines (Leela Chess Zero in 2018).

Analysis of the data will be split in three sections.

Descriptive Statistics:
Visualisation of the oucome variables over time and career of the players

Increase in quality over time (H1):

Linear and nonlinear mixed-effect regression with time (year) as an independent variable and each of the dependent measures (game accuracy, share of optimal moves, centipawns deviation) as a dependent variable. Predictors are the three groups, whose slope of regression would be then compared.

Correlation with chess engine strength (H2):
- Simple Correlation Analysis between the accuracy (and other two dependent variables) and the Elo rating of the strongest chess engine
- Engine Elo as another, time-variate, predictor in mixed effect analysis

Impact of PC and database availability (H3):
- Interrupted Time Series or Step Change Analysis looking for a noticeable jump (inflection point) in the quality of decision-making after the introduction of PCs and databases (around mid-1990s to early 2000).

Impact on juniors and top players vs. seniors (4):
- Differential Interrupted Time Series comparing the magnitude of the change (after introduction of PCs and later neural network engines) between the junior/top players and seniors and adding the group variable in interactions in the H1, H2 and H3 models.

General Considerations (control variables):

- *Total number of games played*
- *Total number of tournaments participated in*
- *Total number of moves*

No files selected

Randomization

No response

Comment on Design Plan:

The sample was the same as in the preregistration. We did not use directly the control variables, but provide the number of individual decisions the averages were based in the OSF data.

Variables

Manipulated variables

Dependent Measures: The quality of decision making will be measured by individual moves, more specifically its quality as compared to the strongest chess engine, Stockfish. Just for a comparison, the best human chess players, Magnus Carlsen, has an Elo rating of 2851 (mean is around 1500, SD is around 300-350 Elo points). The latest version of Stockfish, 16, has an Elo of 3550. This 700-point rating difference corresponds to a win probability of about 99.9% for the open-source engine. This means that Carlsen would have to play about 1000 games against Stockfish to have a chance of winning one game. We will pit every move of every game for a player against the engine benchmark. We will obtain the following measures: 1) Individual games accuracy (see https://lichess.org/page/accuracy) 2) Share of optimal moves (i.e. moves chosen by the engine too) in individual games 3) Centipawns deviation from the optimal move (how much worse is the chosen human move as measured in pawns, value 1, from the engine move) for each move in individual games. These measures will be averaged across years (global comparison) and across years for each player in the dataset (local/player comparison). In other words, all moves and games in a certain year or in a certain year for a certain player will be averaged to get a single value for 1) average game accuracy in that year, 2) average share of optimal moves (defined as all moves within 0.5 pawns of evaluation), 3) average of centipawns for all moves, and 4) count of inaccuracies, mistakes, and blunders (the difference between the move played and top engine choice between 0.5 and 1 is an inaccuracy, 1-2 is mistake, and >2 blunder) Independent Measures. In addition to the already mentioned the cohort variable (i.e. junior, top, and senior players), we will have two other factors. The first will be the highest Elo rating of chess engine in particular year, starting from 1985, the same year we have the data for the dependent measures. The second factor will take into account technological advances throughout the years, and will include the following milestones: a) readily available personal computers (PCs) with strong chess engines and available database of games (around the mid-1990s to early 2000), and b) readily available neural network chess engines (Leela Chess Zero in 2018).

No files selected

Measured variables

We will pit every move of every game for a player against the engine benchmark. We will obtain the following measures: 1) Individual games accuracy (see https://lichess.org/page/accuracy) 2) Share of optimal moves (i.e. moves chosen by the engine too) in individual games 3) Centipawns deviation from the optimal move (how much worse is the chosen human move as measured in pawns, value 1, from the engine move) for each move in individual games. These measures will be averaged for each player, for the year of interest. In other words, all moves and games in a certain year of a certain players will be averaged to get a single value for 1) average game accuracy in that year, 2) average share of optimal moves, 3) average of centipawns for all moves, and 4) count of inaccuracies, mistakes, and blunders.

No files selected

Indices

No response

No files selected

Comment on Variables:

We use the same manipulated variables as stated in the preregistration. We only added Change Win % (from the optimal computer move) as this has become a way of establishing decision making quality in chess.

Analysis Plan

Statistical models

Analysis: The analysis will consist of: 1. Visualisations of the dependent variables over the years and players' careers. 2. Linear and nonlinear mixed-effect regressions with time (year) as an independent variable and each of the dependent measures (e.g. game accuracy, share of optimal moves, centipawns deviation, inaccuracies/mistakes/blunders) as a dependent variable. Predictors are the three groups, whose slope of regression would be then compared and engine Elo, as well as their interactions. 3. Simple Correlation Analysis between the accuracy (and other two dependent variables) and the Elo rating of the strongest chess engine 4. Interrupted Time Series or Step Change Analysis looking for a noticeable jump (inflection point) in the quality of decision-making after the introduction of PCs and databases (around mid-1990s to early 2000) and after the introduction of Leela Chess Zero in 2018. 5. Differential Interrupted Time Series comparing the magnitude of the change (after the introduction of PCs and databases in mid-1990s and Leela Chess Zero in 2018) between the junior/top players and seniors. 5. Case study analysis: Magnus Carslen, the top player in the last 15 years or so, gameplay and changes in rating over his career, specifically, any sudden improvements after the introduction of Leela Chess Zero in 2018. General Considerations (control variables): - Total number of games played - Total number of tournaments participated in - Total number of moves

No files selected

Transformations

No response

Inference criteria

No response

Data exclusion

Moves of each game for the beginning (1 to 10) and end (from 60+) will be removed from analysis as in previous research as they are either too accurate and bring little information (first moves) or generally too difficult compared to engine perfect play (endgames are often solved).

Missing data

No response

Exploratory analysis

No response

Other

Other

No response

Comment on Analysis Plan:

We use the same manipulated variables as stated in the preregistration. We used GAMs instead of LMM/HLM because they captured the nonlinear nature in the data markedly better. LMM models could not converge with when cubic terms were used.
